# Reducing health inequalities with interventions targeting behavioral factors among individuals with low levels of education - A rapid review

**DOI:** 10.1371/journal.pone.0195774

**Published:** 2018-04-16

**Authors:** Andreas Vilhelmsson, Per-Olof Östergren

**Affiliations:** Division of Social Medicine and Global Health, Department of Clinical Sciences Malmö, Lund University, Malmö, Sweden; Universidad del Desarrollo, CHILE

## Abstract

Individuals with low levels of education systematically have worse health than those with medium or high levels of education. Yet there are few examples of attempts to summarize the evidence supporting the efficacy of interventions targeting health-related behavior among individuals with low education levels, and a large part of the literature is descriptive rather than analytical. A rapid review was carried out to examine the impact of such interventions. Special attention was given to the relative impact of the interventions among individuals with low education levels and their potential to reduce health inequality. Of 1,365 articles initially identified, only 31 were deemed relevant for the review, and of those, nine met the inclusion and quality criteria. The comparability of included studies was limited due to differences in study design, sample characteristics, and definitions of exposure and outcome variables. Therefore, instead of performing a formal meta-analysis, an overall assessment of the available evidence was made and summarized into some general conclusions. We found no support for the notion that the methods used to reduce smoking decrease inequalities in health between educational groups. Evidence was also limited for decreasing inequality through interventions regarding dietary intake, physical activity and mental health. Only one study was found using an intervention designed to decrease socioeconomic inequalities by increasing the use of breast cancer screening. Thus, we concluded that there is a lack of support regarding this type of intervention as well. Therefore, the main conclusion is that solid evidence is lacking for interventions aimed at individual determinants of health and that more research is needed to fill this gap in knowledge.

## Introduction

Many governments currently embrace the notion that public health policy should address not only health itself but also its social determinants. For instance, educational attainment (or a lack thereof) is one important social determinant of health (SDH) and a useful indicator for socioeconomic status (SES), which also encompasses income, wealth, power, social class, etc. [1]. This may be explained by factors such as higher incomes among educated people, as well as higher levels of general literacy, which allow more educated individuals to make better-informed health-related decisions [2].

Positive general trends over time in major social determinants of health (e.g., increasing income and educational attainment) have been associated with increasing inequality in health between different socioeconomic groups [1]. This is also true for a welfare state like Sweden, where life expectancy has been increasing for people of all educational levels. However, this increase has lately been more prominent for people with high education, whereas women with only compulsory school (i.e., 9 years of education) have experienced the least improvements [3].

Usually, two strategies are often distinguished when discussing public health policy interventions that address single determinants of health in the context of reducing health inequalities: targeted interventions vs. population-wide interventions. Both strategies could aim at closing the health gap between worse-off groups and the rest of the population, but only population-wide interventions can address the health gradient across the whole population [1]. Whether a marginalized group or the whole population is targeted depends partly on the interpretation of the underlying mechanisms of social inequalities in health. The population-wide approach is usually preferred if the mechanisms are perceived to affect all social strata. However, if the mechanisms are primarily perceived to affect individuals at the low end of the socioeconomic spectrum, targeted interventions are preferred [4].

Population-wide interventions targeting individual behavior, however, may have no impact on health inequalities and could even risk exacerbating them, since their impact is often more prominent among those who have more resources, such as higher levels of education [5]. For example, many effective interventions for smoking cessation are more readily adopted by individuals with high educational levels, thus leading to greater health inequalities caused by smoking [6–7]. In contrast, targeted interventions by definition have an impact among less privileged or vulnerable groups exclusively [8–9], but targeting individuals with low educational levels might be counterproductive due to perceived stigmatizing effects or paternalistic connotations regarding this approach.

For that reason, it is not sufficient for policymakers who wish to reduce health inequalities to simply assume that they will accomplish this goal if they only have information that an intervention is effective overall, without considering the possible differential effects regarding education level. However, there are few published examples of meta-analyses or reviews of empirical studies regarding the differential effects of different intervention strategies to reduce health inequalities by addressing individual health behaviors [10]. Our study was performed to contribute to help fill in this knowledge gap.

In Sweden, there has been a public health debate about the most efficient way to reduce health inequalities between educational groups. This review was conducted for the Public Health Agency of Sweden to assess the magnitude of evidence regarding intervention evaluations with high-quality designs concerning health-related behavior, which have shown a higher impact among individuals with a low educational level, as well as the potential of reducing health inequality.

## Methods

Rapid reviews use streamlined traditional methods for systematic review to help synthesize and communicate evidence within a shortened time frame [11]. The review was completed within four months and was conducted according to the PRISMA guidelines (Preferred Reporting Items for Systematic Reviews and Meta-Analysis [12]).

### Search strategy

We conducted database searches between November 2014 and February 2015 on PubMed (Medline), Embase, CINAHL, SocINDEX, PsycINFO, and Web of Science for the years 1990–2015. Relevant articles for inclusion were identified using the search term “health status” combined with MESH terms “health promotion”, “educational status”, text words “intervention” and “prevention”, and an isolated search on the MESH term “social determinants of health” (see also S1 Appendix). Reference lists of key papers were searched, including cited grey literature such as reports, dissertations and working papers. However, this did not generate any articles beyond those found in the initial database searches. The combined search strategy for CINAHL, PsycINFO, and SocINDEX was (MH”health status+” OR “health promotion”) AND “educational status” AND (interventions OR prevention).

The search strategy for Medline used MESH terms and textword searching combining terms as follows: “health promotion”[Mesh]) AND “educational status”[Mesh] AND intervention [Text Word]. A separate search on Medline for the MESH term “social determinants of health” was also conducted. Finally, a search was done on Embase for the search terms “educational status” AND “health promotion” AND (interventions OR prevention). Full details of the electronic searches are available as an electronic supplementary file (S1 Appendix).

### Study selection

The database searches yielded 1,929 articles. After duplicates were removed, 1,365 articles remained. One of the authors (AV) then screened all article titles and abstracts using inclusion criteria to determine which studies were relevant. Eligible for inclusion were: (1) studies with evaluations of non-healthcare-based interventions regarding health-related behavioral factors among different educational groups. Further, these should have been published in English in international peer-reviewed scientific journals between 1990 and 2015 from countries with developed welfare systems (i.e., from countries in Europe, North America, Australia, and New Zealand); (2) studies comparing those receiving the intervention with a control group and thereby applying one of the following study designs: randomized controlled trials or non-randomized trials with a cohort design (in the case of community interventions, a control area was required); (3) studies where the intervention included at least 100 individuals.

The main reason for exclusion was that the intervention did not measure educational status (n = 812). Studies were also excluded for not having original data (n = 169) or for having outcomes (individual health-related behavior) that did not conform to our study aim (n = 160). Some studies did not contain an intervention (n = 94), and there were studies included in the search terms that were unrelated to the study objective (n = 99).

**Fig 1:**
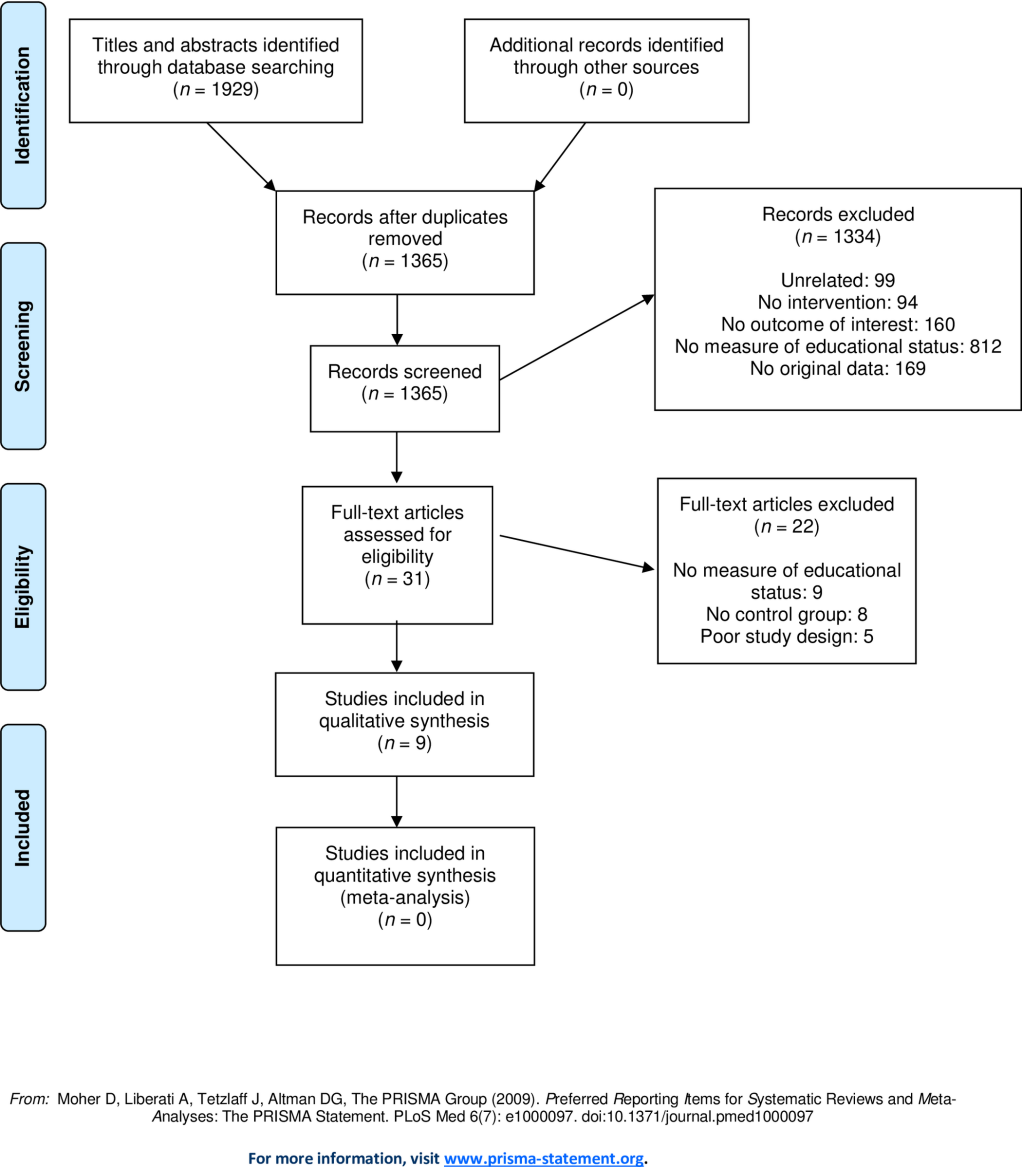
Flow chart of the literature search and screening process.

Thus, of the 1,365 articles, 1,334 were excluded in the first screening process, which generated 31 articles. The full text of these articles were assessed independently by both authors (AV and P-OÖ) while applying the inclusion criteria with more detailed information, as well as quality criteria according to guidelines used by the Swedish Agency for Health and Technology Assessment and Assessment of Social Service, a public national agency in charge of providing impartial and scientifically reliable information to decision makers and health care providers [13]. The following aspects of quality were assessed: (i) confirmation of appropriate study design, (ii) possible selection bias due to sample recruitment procedures, (iii) use of validated measures regarding both exposure and outcome assessment, and (iv) reasonable control for confounding factors, such as age and gender imbalance between intervention and non-intervention groups. Differences in the primary judgment between the authors was resolved by discussion and consensus. Of the 31 articles left at this stage of the analysis, 22 were excluded for lacking an adequate measure of educational status (n = 9) or proper control group (n = 8). Five articles were excluded for lacking an appropriate study design, leaving nine articles for the final analysis (Fig 1 and S1 Table).

We concluded that we could not perform a formal meta-analysis because of different study designs, study settings, and quality of analysis. Furthermore, outcomes were not measured in ways that permitted comparison across the included studies. Therefore, we were limited to making an overall assessment of whether the evaluations of the interventions’ impacts yielded results indicating that inequalities in health could be reduced between groups and individuals with different educational backgrounds.

## Results

Of the nine publications included, all applied prospective study designs. Five studies were randomized controlled trials [14–18], three were community interventions [19–21], and one was a quasi-experimental trial [22] (Table 1). Seven studies evaluated a targeted approach [14–16,17–19,22], and two evaluated a population-wide approach [20,21].

**Table 1:** Characteristics of studies included in the review.

Reference	Study design	Setting	Sample	Intervention	Health outcome	Results	Effect on SDH
Stanczyk et al. (2013) [14]	RCT	Netherlands	Smokers >16 years of age who were motivated to quit within the following six months - N = 139 - Mean age: 47,39 years - Educational level n (%) -Low (n = 68, 48.9%) -High (n = 71, 51.1%)	Web-based tailored intervention: (n = 74 to text condition and n = 65 to video condition) completed the first session of the web-based tailored intervention and were subsequently asked to fill out a questionnaire assessing message-processing mechanisms and future adherence.	- Processing mechanism - Future adherence	- No interaction effects between delivery strategies (video vs. text) and educational level on message-processing mechanisms and future adherence. - Lower educated participants showed higher attention (F_1,138_ = 3.97; P = .05) and processing levels (F_1,138_ = 4.58; P = .04). - Lower educated participants were more inclined to visit the computer-tailored intervention website again (F_1,138_ = 4.43; P = .04).	Moderate
Cantrell et al. (2013) [19]	Community intervention	USA	U.S. adult smokers from diverse racial/ethnic and SES groups recruited from two online research panels - N = 3,371(n = 3,371) - Mean age: 44,17 years	Web-based experimental study to view either pictorial health warning labels (n = 1,706) or text-only warnings (n = 1,665) found on tobacco packaging. Participants viewed the labels and reported their reactions.	- Intention to quit smoking. - Salience - Perceived impact - Credibility	- Pictorial health-warning message gave significant stronger reactions for each outcome salience. - No significant results were found for interactions between condition and education. - Intention to quit: mean % for low education 35.5% for text and 39% for text+pictorial vs. 47.4% and 49.8% for high education.	Weak - Risk exacerbating inequalities in health between educational groups.
Wendel-Vos et al. (2009) [20]	Community intervention	Netherlands	Men and women aged 31–70 years living in and around certain areas in the Netherland. - N = 3114	Community-based health intervention (Hartslag Limburg) aimed at preventing cardiovascular disease by comparing 5-year mean changes in lifestyle factors (1998–2003), between subjects from the intervention area (n = 2,356) and the control area (n = 758) and between men and women and for those with a low, moderate and high education. 50% of intervention took part in deprived areas	- Energy intake in terms of fat intake - Time spent on leisure-time - Walking - Bicycling - Sports - Smoking behavior	Among those with a low education level, a significant difference (p≤0.05) between the intervention region and the control region were found for: - Fat intake: −3.0 (g/d) for low educational level vs. −1.7 (g/d) for high educational level. - Time spent walking +2.3 (hrs/wk) for low educational level vs. +0.9 for high educational level. - Time spent bicycling +0.6 (hrs/wk) vs. −0.3 for high education (p≤0,05). - Smoking: among intervention 6,2% with low educational level quit smoking vs. 6.1% among high educational level	Weak - Too small differences to draw any conclusions.
Øverby et al. (2012) [21]	Community level intervention	Norway	Sixth- and seventh-grade pupils from 27 Norwegian elementary schools completed a questionnaire in 2001 (n = 1,488) together with sixth- and seventh-grade pupils from the same schools that completed the same questionnaire in 2008 (n = 1,339).	The project “Fruits and Vegetables Make the Marks” a national free school fruit program 2001–2008 with focus on SES	- Consumption of unhealthy snacks (soda, candy and potato chips)	The frequency of unhealthy snack consumption decreased and was more pronounced in schools that had been included in the national free school fruit program (−2.8 times/week). Effect was significant (p = 0,004) in reducing the frequency of unhealthy snack consumption in children of parents without higher education (−3.8 times/week).	Moderate
Cameron et al. (2014) [15]	Cluster-randomized controlled trial	Australia	542 mother/infant pairs from existing 62 first-time parent groups. - N = 389 first-time mothers and infants tested for maternal education - Mean 32.3 years for mothers	The Melbourne Infant, Feeding, Activity and Nutrition Trial (InFANT) 2008–2010 involving 6 × 2-hour dietarian-delivered sessions, DVD and written resources from infant age 4–15 months was assessed by comparing an intervention group (n = 191) with a control group (n = 198)	- Infant diet (3 × 24h diet recalls) - Physical activity (accelerometry - Television viewing - Body mass index, BMI	- BMI change −0.07 (p = 0.49) for low educational level vs. 0.04 for high educational level - Vegetable intake (g/d) 14.79 (p = 0.23) for low educational level vs. 8.65 for high educational level - Water intake: 65,35 g (p = 0,02) for low educational level vs. −6.16 for high educational level - Non-core drinks intake (g/d) 5.3 (p = 0.49) for low educational level vs. −7.67 for high educational level - Sweet snacks intake (g/d) −1.55 (p = 0.43) for low educational level vs. −5.22 for high educational level - Savory snacks intake (g/d) −1.17 (p = 0.35) for low educational level vs. −0.83 for high educational level - Television viewing (min/d) −19.43 (p = 0.01) for low educational level vs. −13.39 for high educational level - Physical activity (min/d) 1.78 (p = 0.77) for low educational level vs. −5.87 for high educational level	Weak - Risk of exacerbating inequalities in health between educational groups.
Vander Ploeg et al. (2014) [22]	Quasi-experimental trial	Canada	Grade five school students (n = 412) from 10 invited school in 2009, with follow-up in 2011 (n = 339) compared to 20 control schools (n = 845 students) with follow up in 2011 (n = 680)	Comprehensive School Health (CSH) program implemented in schools located in socioeconomically disadvantaged neighborhoods. The Alberta Project Promoting active Learning and healthy Eating in Schools (APPLE Schools). SES was determined from parent self-report. Low-active, active, and high-active children were defined according to step-count tertiles. Grade five students included in analysis in 2009 (n = 198) with follow up in 2011 (n = 196) were compared with controls in 2009 (n = 450) and at follow-up in 2011 (n = 300)	- Physical activity	From 2009 to 2011, children within the low-education groups from intervention schools experienced increases in physical activity (+23,8%) and 23.6% greater than children within these groups from comparison schools, respectively	Moderate
Van der Waerden et al. (2013) [16]	RCT	Netherlands	Low SES women (20–55 years) with elevated stress or depressive symptoms levels - N = 161 - Mean age: 43.9 years	Study subjects randomly assigned to the combined exercise/psycho-education (EP, n = 55), exercise only (E, n = 46), or a waiting list control condition (WLC, n = 48) with postponed intervention four months later. Follow up at 2, 6 and 12 months	- Depressive symptoms (CES-D) - Perceived stress symptoms (PSS)	Both interventions (E + EP) among women with the lowest educational level had lower PSS at post-test than women from control condition: - E = -4,13 (p = 0.02, effect size = 0.35) - EP = -4,12 (p = 0.024, effect size = 0.53)	Moderate - Important that the intervention target those with low educational level
DeSocio et al. (2013) [17]	RCT	USA	Unmarried adolescent (n = 429) mothers <19 years of age were randomly assigned to an intervention and a control group. The participants met at least two of three criteria for social disadvantage: (a) Unmarried (b) <12 years of education (c) Unemployed - Mean age: 18 years (range 12–33)	The Memphis New Mothers’ Home Visitation Program 1990–1991. The intervention group (n = 132) received free transportation and developmental screening plus intensive nurse home visitation through their infants’ second birthdays, in total 57 nurse visits.The control group (n = 297) received both free transportation and developmental screening/referral for their children at 6, 12, and 24 months of age.	- Self-agency	Adolescent mothers with lower cognitive ability who were behind their age-appropriate grade level in school made the greatest self-agency gains (p <0,003)	Weak - Could however be a way to reach those with low educational level
Kreuter et al. (2010) [18].	RCT	USA	African American women (n = 489) age ≥40 and never diagnosed with breast cancer were recruited from low-income neighborhoods. - Mean age: 61.1 years	Recruited women were randomly assigned to narrative video (n = 244) or informational video (n = 245) Telephone follow-up interviews at 3 and 6 months post-baseline	- Use of mammography	Use of mammography at 6-month follow-up did not differ for the narrative vs. informational groups, but one exception was among women with low educational level (65% vs. 32%, p < .01). For women with high educational level this was not seen (42,1% vs. 43,1%, p = 0,91)	Weak - Could however be a way to reach those with low educational level

The sizes of the study samples varied between 161 and 3,371 participants included in interventions from five different countries: three from the Netherlands [14,16,20], three from the USA [17–19], one from Australia [15], one from Canada [22], and one from Norway [21] (Table 1). Two interventions occurred within school settings [21–22], two were web-based [14,19], two were implemented within a public health service setting [16,20], one involved a home-nurse visitation service [17], and one was in a maternal healthcare setting for first-time parent groups [15], and another one was in a mobile health communication research facility [18] (Table 1).

### Intervention approaches for reducing inequalities in health

The included study results could be divided into five different health outcomes: three studies were interventions for smoking cessation [14,19–20], three evaluated interventions for better dietary intake [15,20–21], two were interventions for increasing physical activity [20,22], two were interventions for better mental health [16–17], and one study comprised an intervention investigating changes in use of mammography [18] (Table 1). Only one of the included studies explicitly set out to evaluate the intervention according to its effect on inequalities in health [22] (Table 1).

### Findings as reported by the authors

#### Smoking cessation

Three studies evaluated different tobacco control interventions with comparisons of the impact between individuals with different educational background [14,19–20]. Stanczyk et al. [14] investigated whether differences exist in message-processing mechanisms and future adherence by assigning Dutch smokers >16 years of age to receive either video or text-based messages in a tailored web-based intervention. No interaction effects were found between delivery strategies (video vs. text) and educational level on message-processing mechanisms and future adherence to participation in the intervention program. However, in both groups, the results indicated that participants with less education showed higher attention and were more inclined to visit the website again. The intervention never actually investigated whether individuals manage to stop smoking because of the intervention, so it could not be concluded whether it actually works in a real-life setting.

Cantrell et al. [19] evaluated the potential impact of warning labels among adult smokers in the U.S. from diverse racial/ethnic and socioeconomic subgroups. The participants were recruited from two online research panels into a web-based experimental study to view either pictorial health warning labels or text-only warnings found on tobacco packaging. Overall, the pictorial message had significantly stronger effects for each outcome. However, no significant results were found for interactions between intervention mode and education. The only exception concerned the outcome of intention to quit, where the interaction between intervention mode and education was nearly significant. However, individuals with high educational levels were more inclined to quit smoking in the next 30 days.

In the third study, Wendel-Vos et al. [20] evaluated the net effect of a Dutch cardiovascular disease-prevention program (Hartslag Limburg) on lifestyle factors after 5 years of intervention. Change in smoking behavior was one outcome, and the study compared the intervention’s impact among subjects from the intervention area and the control area, as well as differences in impact between men and women and between those with low, moderate, and high educational levels. However, no change in smoking was observed between educational groups regarding the tendency to quit smoking.

#### Interventions for better dietary intake

The aforementioned Hartslag Limburg Intervention [20] also evaluated changes in energy and fat intake. Differences between the intervention group and the control group were statistically significant among those with low educational levels regarding energy intake and fat intake. However, the difference was too small compared to that among those with a moderate or high educational levels to draw any secure conclusions regarding an impact on health inequalities.

Øverby et al. [21] evaluated whether the introduction of a school fruit program in Norwegian schools was associated with a reduced frequency of consuming unhealthy snacks with respect to sex and SES among seventh-grade students from Norwegian elementary schools who completed a questionnaire in 2001 and 2008. The frequency of unhealthy snack consumption decreased, and the decrease was largest in the schools that had been included in the national free fruit school program. The effect was significant in reducing the frequency of unhealthy snack consumption among children whose parents had low educational levels.

Cameron et al. [15] evaluated an intervention for early childhood obesity prevention among first-time parents according to the mothers’ educational level and age. The mothers were randomly allocated to an intervention or a control group. The intervention involved dietician-delivered sessions as well as DVD and written resources for mothers whose infants were 4–15 months old. The impact of the intervention was overall more effective among mothers with a high educational level. Only the outcome of water consumption was greater in infants whose mothers had low educational levels.

#### Interventions to increase physical activity

The Hartslag Limburg Intervention [20] also investigated time spent on physical leisure activities (walking, bicycling, and sports) for those with low, moderate, and high education levels. Among those with a low educational level, there was a significant difference between the intervention group and the control group for time spent walking and time spent bicycling. However, the change was too small compared to those in the moderate or high education groups to draw any conclusions regarding the potential of the intervention to reduce inequalities between educational groups.

Vander Ploeg et al. [22] compared the two-year changes in physical activity among 10 to 11-year-old children attending Canadian schools with or without health promotion programs by activity level, body weight status, and SES to assess whether health promotion programs could reduce or exacerbate health inequalities. Pedometer and demographic data were collected from cross-sectional samples of fifth-grade children from intervention and comparison schools. SES was determined from parents’ self-reports. Children within the low-education and low-income groups from the intervention schools experienced greater increases in physical activity than comparable children from comparison schools. These increases were more pronounced than those observed among children whose parents had high educational level and high incomes.

#### Interventions for better mental health

Van der Waerden et al. [16] evaluated an intervention that aimed to reduce stress and depressive symptoms in low-SES Dutch women (20–55 years) with elevated stress or depressive symptoms levels. The women were randomly assigned to a combined exercise/psycho-education (EP), exercise only (E), or waiting list control group. Depressive symptoms and perceived stress were measured before and immediately after the intervention and at 2, 6, and 12-month follow-up. Women in the EP and E groups with the lowest educational levels reported more stress reduction in post-test assessments than women with higher educational levels. Furthermore, analyses using a perceived stress scale, showed that women from the EP and E groups with the lowest educational level had lower perceived stress scores at the post-test assessment than women from the control group.

DeSocio et al. [17] analyzed data from unmarried adolescent mothers <19 years old who were randomly assigned to intervention and control groups. They examined factors that influence self-agency change over their time of involvement in a home visitation program. Women in the control group received both free transportation and developmental screening/referrals for their children at 6, 12, and 24 months of age. Women in the intervention group received the same benefits plus an intensive home visitation by a nurse, comprising 57 nurse visits in total. Adolescent mothers with lower cognitive ability who were behind their age-appropriate grade level in school made the greatest gains in self-agency.

#### Intervention investigating changes in a mammography program participation

Kreuter et al. [18] evaluated an intervention that aimed to increase the use of mammography. African American women ≥40 old who were never diagnosed with breast cancer were recruited from low-income neighborhoods. Participants were randomly assigned to watch a narrative video comprising stories from African-American breast-cancer survivors or a content-equivalent informational video that used a more expository and didactic approach. The impact of the intervention was measured immediately post-exposure and at 3 and 6 months by telephone follow-up interviews. The use of mammography at the 6-month follow-up did not differ between the narrative and informational groups overall, but it did differ among women with less than a high school education, who had higher mammography rates.

## Discussion

This rapid review identified nine studies that evaluated interventions targeting health-related behavior, while taking educational inequalities into account. Five different health outcomes were analyzed: smoking, dietary intake, physical activity, mental health, and mammography.

Given the heterogeneity of the studies included, we could not perform a meta-analysis because of the different study designs, study settings, and quality of analysis. Furthermore, outcomes were not measured in ways that permitted comparison across the included studies. Therefore, we were limited to making an overall assessment of whether the evaluations of the intervention impacts yielded results indicating that inequalities in health could be reduced between groups and individuals with different educational backgrounds. Regarding interventions aiming at smoking cessation, we could not draw any decisive conclusions. Of the three studies found, only one [14] provided some support for benefiting those with lower education. We also found limited evidence for decreasing inequality through interventions regarding dietary intake [21], physical activity[22], and mental health [16–17]. The Norwegian school fruit intervention is particularly interesting since this program seems feasible to implement as a cost-effective health-promoting initiative. Since we only found one single study attempting to increase the use of breast cancer screening (mammography) [18], we concluded that there is not enough scientific evidence concerning the potential for increased health equity for this approach.

All in all, there are few intervention evaluations with high-quality designs regarding health-related behavior, which have shown a higher impact among individuals with a low educational level. Our results are in line with previous research that used less strict quality criteria for including studies. That research found limited evidence of effective community-based interventions, such as interventions aimed at diet and physical activities for socio-economically disadvantaged groups [23]. As previously mentioned, policies associated with positive developments in average public health have also been associated with increased inequality in health outcomes between different socioeconomic groups [1,3]. This may be due to high-SES individuals benefitting more from such interventions because of greater access to resources (such as education), which positively interact with the intervention [5,6–7]. In fact, two of the studies included in our review were interventions where the health-promoting initiative risked exacerbating inequalities in health instead of reducing them [14,19]. Nevertheless, we do not argue in favor of a nihilistic position regarding public health interventions concerning individual determinants of health. As suggested by the CSDH and many others, the underlying issue of health inequity is the systematic generation of a socially unjust distribution of individual level resources, such as money, power, and resources depending on elements of the social structure [24].

The likelihood of public-health interventions having a higher impact on health inequality could be increased by designing them to be embedded in the public welfare system, which has essentially been developed to reallocate money, power, and resources. A prominent example of this is the early interventions in terms of mother and child health services, which were developed in Scandinavian countries in the 1930s [25–26]. These services include an important element of resource transfer to individuals and families with low SES and were embedded in other elements of the welfare state, such as progressive taxation and gender equity policies [27]. A more recent example is the taxation of sugar-sweetened beverages or “soda tax” as it is sometimes referred to [28–29] which after its introduction in Mexico has resulted in a more pronounced decline of high-energy foods in low SES households.

Moreover, such embedded interventions should have greater potential to address the issue of the gradient of inequality across the spectrum of socioeconomic statuses rather than just the gap between the worst-off and the rest of the population [30]. Hence, the gradient in health inequalities could be efficiently addressed by the strategic principle of proportionate universalism [31], which involves a common integrated system of health and welfare services that serves the whole population according to need and is financed according to ability.

### Limitations

Our study has several limitations. We chose the search words to include a wide range of studies given the broad nature of the focus. However, we still cannot not rule out that we could have missed relevant articles since they could be classified by key words that highlight other dimensions of the article content. To compensate for this, manual searches were also conducted, and searches were repeated several times and in consultation with an information specialist at the university library.

The most important limitation is the small number of studies that remained at the end of our selection procedure, which was the result of our choice not to compromise with the inclusion and quality criteria. Our selection criteria (interventions targeting individuals, the individual approach, and specific focus on low education groups) was limited as a report commissioned by the Public Health Agency of Sweden focusing on these aspects. The fact that one of us screened all titles and abstracts due to the limited time frame is another limitation of our study. However, we regard this observation as one of the outcomes of the study, which we hope will provoke further discussion regarding the common use of this type of methodology for generating the evidence base for policy recommendations regarding public health. These limitations made our original aim difficult to attain by means of more formal types of analysis, which forced us to change our strategy by acknowledging that the limited evidence base itself was the main finding.

## Conclusions

There are very few studies of high quality design that have examined the potential for interventions aimed at individual determinants of health to reduce health inequalities. This review could only identify nine intervention studies with limited comparability. Therefore, the main conclusion is that solid evidence is lacking for the mentioned interventions and that more research is needed to fill this gap in knowledge. However, some potentially promising interventions were identified, which merit further research.

## Supporting information

S1 Appendix Database searches. (DOC)

S2 Appendix PRISMA 2009 checklist. (DOC)

S1 Table Excluded studies. (DOC)
