# Supplementary material for: Reducing health inequalities with interventions targeting behavioral factors among individuals with low levels of education - A rapid review
Source: PLoS One. 2018 Apr 16;13(4):e0195774. doi: 10.1371/journal.pone.0195774 (PMC5901784; doi:10.1371/journal.pone.0195774)
Supplement: S1 Appendix — (DOC) [file pone.0195774.s001.doc]

S1. Appendix. Database searches

**Literature search 1**

CINAHL, PsycINFO, SocINDEX in February 2015.

| **Search terms** | | **Hits** |
| --- | --- | --- |
| **1** | “educational status” | 25,351 |
| **2** | (MH "Health Status+") | 59,293 |
| **3** | “health promotion” | 43,535 |
| **4** | *2 OR 3* | *100,456* |
| **5** | *1 AND 4* | *4225* |
| **6** | (interventions OR prevention) | 491,443 |
| **7** | **5 AND 6** | **697** |

Included in search nr. 5 are:

(MH ”Health status+” OR “health promotion”) AND “educational status” (= 4225)

(MH "Health Status+") including searches on:

- Health Status Disparities
- Functional Status
- Mental Status
- Nutritional Status
- Social Determinants of Health

**Literature search 2**

PubMed in February 2015.

| **Search terms** | | **Hits** |
| --- | --- | --- |
| **1** | "Health Promotion"[Mesh]) | 55,649 |
| **2** | "Educational Status"[Mesh] | 40,445 |
| **3** | *1 AND 2* | *697* |
| **4** | Intervention [Text Word] | 373,113 |
| **5** | **3 AND 4** | **127** |

**Literature search 3**

Embase in February 2015.

| **Search terms** | | **Hits** |
| --- | --- | --- |
| **1** | “educational status” | 42,821 |
| **2** | “health promotion” | 97,289 |
| **3** | *1 AND 2* | *1459* |
| **4** | (interventions OR prevention) | 2 004,249 |
| **5** | **3 AND 4** | **641** |

**Literature search 4**

PubMed in February 2015.

| **Search terms** | | **Hits** |
| --- | --- | --- |
| **1** | "Social Determinants of Health"[Mesh]) | 222 |
