# Supplementary material for: Reducing health inequalities with interventions targeting behavioral factors among individuals with low levels of education - A rapid review
Source: PLoS One. 2018 Apr 16;13(4):e0195774. doi: 10.1371/journal.pone.0195774 (PMC5901784; doi:10.1371/journal.pone.0195774)
Supplement: S1 Table — (DOC) [file pone.0195774.s003.doc]

**S1. Table. Excluded studies**

| **Author, Year, Reference, Country** | **Study-design** | **Reason for exclusion** | **Main conclusions** |
| --- | --- | --- | --- |
| Berger-Jenkins et al [1] 2014, US | Longitudinal cohort | Did not have educational status of parents/children as a variable. Too small control group | Comprehensive school-based obesity prevention programs that utilize a public health approach and target students, adults, and communities may be effective at improving nutrition and physical activity knowledge, attitudes, and behavior in large, under resourced public elementary schools in underserved Hispanic communities. |
| Jones et al [2] 2015, US | Method study | Low study quality | We identified fair validity and substantial reliability of a brief, 16-item questionnaire used to access physical activity among low income overweight women. |
| Patel et al [3] 2012, US | Cohort study | No control group | The results of this study demonstrate the feasibility and efficacy of an academic institution collaborating with the African American community to develop a successful prostate cancer educational intervention, an approach that can be expanded to other cancers and other chronic diseases. |
| Marcus et al [4] 2009, Sweden | Cluster RCT | Did not have educational status as a variable. | A school-based intervention can reduce the prevalence of overweight and obesity in 6 to 10-year-old children and may affect eating habits at home. The effect of the intervention was possibly due to its effect on healthy eating habits at school and at home rather than on increased levels of physical activity. |
| Epstein et al [5] 2008, US | RCT | Did not have educational status as a variable. | Reducing television viewing and computer use may have an important role in preventing obesity and lowering BMI in young children, and these changes may be related more to changes in energy intake than to changes in physical activity. |
| Champion et al [6] 2006, US | Prospective randomized intervention study | No control group | These data indicate that tailored approaches are more effective than targeted messages either in print or video format. Another finding of this study is that interactive interventions are effective than non-interactive interventions in increasing adherence and moving African American women forward in the mammogram stage of readiness. |
| Powell and Chaloupka [7] 2009, US | Review | No original data | Limited evidence that small taxes or subsidies will produce significant changes in BMI or obesity prevalence but nontrivial pricing interventions may have some measurable effects on Americans’ weight outcomes, particularly for children and adolescents, low-SES populations, and those most at risk for overweight. |
| Sorensen et al [8] 2003, US | RCT | Did not include educational level, only ‘blue collar’ | Integration of occupational health and safety and health promotion may be an essential means of enhancing the effectiveness of worksite tobacco control initiatives with blue-collar workers. |
| Zoellner et al [9] 2011, US | Quasi experimental design + RCT | No focus on low education | The Community-based participatory research study highlights implementations factors and signifies the community’s active participation in the development and execution of this study. |
| Bergh et al [10] 2012, Norway | RCT | No focus on low education | Social support from teachers might be a potential mediator of physical activity change, and that overweight adolescents might be in need of specially targeted interventions to avoid reducing their enjoyment of physical activity. |
| Zhu et al [11] 2010, US | Methodological study | No original data; Methodological study | The California tobacco control program on smoking prevalence is effective with both low and high education. |
| Ryan et al [12] 2011, UK | Longitudinal cohort study | SES defined as area not individually. No control group; before and after design | Pedestrians in lower SE areas appear less likely to climb stairs than pedestrians in high SE areas. Nevertheless, a stair climbing intervention was equally effective in both areas. |
| Weber Cullen et al [13] 2010  USA | RCT | Did not have educational status as a variable. | The findings in this study document positive outcomes from attaining behavior change goals associated with the psychosocial and environmental influences on food choice behavior. |
| Weber Cullen et al [14] 2009  USA | RCT | Did not have educational status as a variable. | Expanded Food and Nutrition Education Program programs in local communities could have a significant impact on family dietary behaviors for populations at risk of obesity. |
| Magnusson et al [15] 2011 Sweden | Ecological longitudinal study | No control group; before and after design | Positive changes in diet and weight status were observed, especially in girls, within a low-income multi-ethnic community undergoing a health promotion intervention. Our result underscore the multi-factorial etiology of childhood obesity and the importance of continuing tailored, gender-sensitive prevention efforts. |
| Durkin et al [16] 2009, US | Longitudinal study | No real intervention or control group; before and after design | Emotionally evocative ads and ads that contain personalized stories about the effects of smoking and quitting hold promise for efforts to promote smoking cessation and reduce socioeconomic disparities in smoking. |
| Pickett et al [17] 2005 US | Population based cohort study | No control group | The introduction of an inexpensive, easy, public health intervention has not reduced social inequalities in sudden infant death syndrome; in fact, the gap has widened. |
| Nagelhout et al [18] 2013, Netherlands | Longitudinal study | No control group; before and after design | The Dutch smoking cessation campaign did not succeed in reaching low to moderate educated smokers more than high educated smokers. |
| Niederdeppe et al [19] 2008, US | Longitudinal cohort study | Community intervention without external control group, no focus primarily on low education | Some media campaign messages appear less effective in promoting quit attempts among less-educated populations compared with those who have more education. |
| Jacobs et al [20] 2011, Belgium | RCT | No focus on intervention effects on different educational groups. Only evaluate the effect of the intervention | Six months post-baseline, our results showed that ‘usual care’ was as effective as a cardiovascular prevention program in changing health behavior and BMI in highly educated adults. However, a higher intervention dose led to better responses than a lower dose. The present study highlights the importance of the intervention dose and delivery mode for the development, evaluation, and optimization of health promotion programs. |
| Govil et al [21] 2009, US | Multicenter study | No control group | The observed benefits for coronary heart disease patients with low SES indicate that broadening accessibility of lifestyle programs through health insurance should be strongly encouraged. |
| Durkin et al [22] 2011, Australia | A kind of case control study | No control group | Tobacco control ads that predominately depict highly emotive stories of the consequences of smoking-related diseases on smoker’s lives and the lives of their family and friends are likely to optimize disadvantaged groups’ utilization of quit line services and increase their chances of quitting successfully. |

**References**

1. Berger-Jenkins E, Rausch J, Okah E, et al. Evaluation of a Coordinated School-Based Obesity Prevention Program in a Hispanic Community: Choosing Healthy and Active Lifestyles for Kids/Healthy Schools Healthy Families. American Journal of Health Education 2014; 45(5):261-270.
2. Jones SA, Evenson KR, Johnston LF, et al. psychometric properties of the modified RESIDE physical activity questionnaire among low-income overweight women. Journal of Science and Medicine in Sport 2015; 18:37-42.
3. Patel K, Ukoli F, Liu J, et al. A Community-Driven Intervention for Prostate Cancer Screening in African Americans. Health Education & Behavior 2012; 40(1):11-18.
4. Marcus C, Nyberg G, Nordenfelt A, et al. A 4-year, cluster-randomized, controlled childhood obesity prevention study: STOPP. International Journal of Obesity 2009; 33:408-417.
5. Epstein LH, Roemmich JN, Robinson JL, et al. A Randomized Trial of the Effects of Reducing Television Viewing and Computer Use on Body Mass Index in Young Children. Arch Pediatr Adolesc Med 2008; 162(3):239-245.
6. Champion VL, Springston JK, Zollinger TW, et al. Comparison of three interventions to increase mammography screening in low income African American women. Cancer Detection and Prevention 2006; 30:535-544.
7. Powell LM, Chaloupka FJ. Food prices and obesity: evidence and policy implications for taxes and subsidies. The Milbank Quarterly 2009; 87(1):229-257.
8. Sorensen G, Stoddard AM, LaMontagne AD, et al. A Comprehensive Worksite Cancer Prevention Intervention: Behavior Change Results from a Randomized Controlled Trial (United States). Journal of Public Health Policy 2003; 24(1):5-25.
9. Zoellner JM, Connell CC, Madson MB, et al. H.U.B city steps: methods and early findings from a community-based participatory research trial to reduce blood pressure among african americans. International Journal of Behavioral Nutrition and Physical Activity 2011; 8:59.
10. Bergh IH, Bjelland M, Grydeland M, et al. Mid-way and post-intervention effects on potential determinants of physical activity and sedentary behavior, results of the HEIA study – a multi-component school-based randomized trial. International Journal of Behavioral Nutrition and Physical Activity 2012; 9:63.
11. Zhu S-H, Hebert KK, Wong S, et al. Disparity in Smoking Prevalence by Education: Can We Reduce It? Glob Health Promot 2010; 17(1):29-39.
12. Ryan J, Lyon K, Webb OJ, et al. Promoting physical activity in a low socioeconomic area: Results from an intervention targeting stair climbing. Preventive Medicine 2011; 52:352-354.
13. Weber Cullen K, Thompson DI, Scott AR, et al. The impact of goal attainment on behavioral and mediating variables among low income women participating in an Expanded Food and Nutrition Education Program intervention study. Appetite 2010; 55:305-310.
14. Weber Cullen K, Smalling AL, Thompson D, et al. Creating Healthful Home Food Environments: Results of a Study with Participants in the Expanded Food and Nutrition Education Program. Journal of Nutrition Education and Behavior 2009; 41(6):380-388.
15. Magnusson MB, Sjöberg A, Kjellgren KI, et al. Childhood obesity and prevention in different socio-economic contexts. Preventive Medicine 2011; 53:402-407.
16. Durkin SJ, Biener L, Wakefield MA. Effects of Different Types of Antismoking Ads on Reducing Disparities in Smoking Cessation Among Socioeconomic Subgroups. American Journal of Public Health 2009; 99(12):2217-2223.
17. Pickett KE, Luo Y, Lauderdale DS. Widening Social Inequalities in Risk for Sudden Infant Death Syndrome. American Journal of Public Health 2005; 95(11):1976-1981
18. Nagelhout GE, Crone MR, van den Putte B, et al. Age and educational inequalities in smoking cessation due to three population-level tobacco control interventions: findings from the International Tobacco Control (ICT) Netherlands Survey. Health Education Research 2013; 28(1):83-91.
19. Niederdeppe J, Fiore MC, Baker TB, et al. Smoking-Cessation Media Campaigns and Their Effectiveness Among Socioeconomically Advantaged and Disadvantaged Populations. American Journal of Public Health 2008; 98(5):916-924.
20. Jacobs N, De Bourdeaudhuij I, Thijs H, et al. Effects of a cardiovascular prevention program on health behavior and BMI in highly educated adults: A randomized controlled trial. Patient Education and Counseling 2011; 85:122-126.
21. Govil SR, Weidner G, Merritt-Worden T, et al. Socioeconomic Status and Improvements in Lifestyle, Coronary Risk Factors, and Quality of Life: The Multisite Cardiac Lifestyle Intervention Program. American Journal of Public Health 2009; 99(7):1263-1270.
22. Durkin SJ, Wakefield MA, Spittal MJ. Which types of televised anti-tobacco campaigns prompt more quitline calls from disadvantaged groups? Health Education Research 2011; 26(6):998-1009.
